# Education-related disparities in reported physical activity during leisure-time, active transportation, and work among US adults: repeated cross-sectional analysis from the National Health and Nutrition Examination Surveys, 2007 to 2016

**DOI:** 10.1186/s12889-018-5857-z

**Published:** 2018-07-28

**Authors:** Shaun Scholes, David Bann

**Affiliations:** 1 Health and Social Surveys Research Group; Research Department of Epidemiology and Public Health, University College London, WC1E 6BT, London, UK; 2 Centre for Longitudinal Studies, University College London Institute of Education, London, UK

**Keywords:** Physical activity, Disparities, Socioeconomic, Race/ethnicity

## Abstract

**Background:**

Understanding socioeconomic disparities in physical activity is important, given its contribution to overall population-wide health and to health disparities. Existing studies examining trends in these disparities have focused exclusively on physical activity during leisure-time and have not investigated the potential moderators of socioeconomic disparities in physical activity. Using self-reported data from the US National Health and Nutrition Examination Survey (NHANES) 2007 to 2016 for 29,039 adults aged 20 years and over we examined education-related disparities in overall (total) moderate-to-vigorous intensity physical activity, and in its sub-components, recreational (leisure-time) and non-recreational (active transportation and work) activity. We also examined if education-related disparities in physical activity were moderated by age, gender, and race/ethnicity.

**Methods:**

Logistic regression models were used to evaluate disparities in physical activity according to education group and their moderation across age, gender, race/ethnicity, and time-period.

**Results:**

Overall activity levels (% ≥150 min/week) were highest amongst highly educated adults, yet contrasting education-related disparities were found for recreational and non-recreational activities (active transportation and work), favoring the highest- and lowest-educated groups respectively. Within each domain of activity, associations were moderated by age and race/ethnicity, and by gender for work-based activity. The net result was that education-related disparities in total activity were substantially larger in older adults (*P* < 0.001) and amongst women (*P* < 0.001). For example, the estimated difference in the probability of being active in the highest versus the lowest educational groups was 23.1% (95% CI: 19.1, 27.2) amongst those aged ≥60 years, yet 10.8% (95% CI: 7.1, 14.6) amongst those aged 20–39.

**Conclusions:**

Education-related disparities in physical activity persisted from 2007 to 2016. Our results suggest that understanding and addressing these disparities requires assessment of their multiple domains, and identification of the demographic sub-groups for which the disparities are more or less pronounced.

**Electronic supplementary material:**

The online version of this article (10.1186/s12889-018-5857-z) contains supplementary material, which is available to authorized users.

## Background

Monitoring socioeconomic disparities in physical activity is important, given the contribution of physical activity to overall population-wide health and to health disparities [1, 2]. Systematic reviews have documented that socioeconomic disparities in physical activity exist in high-income countries [3–5], and a smaller number of studies showed that these have persisted across time (e.g., from 2000 to 2009 [6] or 1990–2004 [7] in the United States). The investigation of trends thus requires updating using more recently collected data; it may also yield policy-relevant information on whether public health initiatives to increase physical activity (and reduce its disparities) have been successful at the population level. Furthermore, previous studies have used single physical activity outcomes capturing activity in leisure-time [7] or activity in an unspecified domain/s [6]. Current public health surveillance efforts are therefore limited, since activity occurs across multiple domains (e.g., leisure-time, active transportation, or work)—each domain is likely to have different relations to indicators of socioeconomic status (SES) [4], and each may be separate targets of intervention in order to improve overall activity levels. In addition, potential moderators of socioeconomic disparities in physical activity have not been investigated. Age, gender, and race/ethnicity arguably represent the most important non-modifiable characteristics that may moderate socioeconomic disparities in physical activity, and understanding this may be informative for policy development. Such moderation may be domain-specific. Furthermore, it is unknown whether any moderation of socioeconomic disparities in physical activity has changed over the last decade.

We addressed these limitations using the National Health and Nutrition Examination Survey (NHANES), the only study in the United States (US) with comparable and repeated cross-sectional data on physical activity for recreational and non-recreational (active transportation and work) activities over the 10-year period from 2007 to 2016 [8]. We examined whether education-related disparities in physical activity outcomes were moderated by age, gender, and race/ethnicity, and explored trends over time. We hypothesized that the magnitude of education-related disparities observed in physical activity outcomes would differ by domain, and would be moderated by demographic sub-group. We also hypothesized that these disparities may have increased over time due to concurrent larger increases in the prevalence of obesity [9] and the disproportionate impact of the Great Recession of 2007–09 [10] amongst the lowest educated groups. Education was used as the indicator of SES in the present study, since it remains relevant throughout adulthood, captures possible differences in knowledge about the positive health impacts of physical activity, and tends to have less missing data than indicators such as income. Alternative indicators of SES such as income are also arguably more likely affected by reverse causation or confounding due to ill-health.

## Methods

The study population was adults aged 20 years and older in the five 2-year survey cycles of NHANES between 2007 and 2016 (*N* = 29,039). NHANES is a repeated cross-sectional survey, with information on its design, data collection, and sampling provided extensively elsewhere [11, 12]. It uses a four-stage stratified cluster probability sample design to sample the non-institutionalized civilian US population to obtain results that are nationally representative of the US population. Response rates during the study period ranged from 61% (2015–16) to 79% (2009–10). The CDC National Center for Health Statistics Research Ethics Review Board approved the NHANES study protocol, and all survey participants provided written, informed consent before completing any questionnaires.

Physical activity data was obtained for persons aged 16 years and older during in-person interviews using an adapted version of the Global Physical Activity Questionnaire (GPAQ) [13] from 2007 to 08 onwards, precluding the assessment of longer-term trends [14]. Briefly, the GPAQ assesses the frequency (number of days per week) and duration (amount of time spent on a typical day) of physical activity undertaken for a minimum of 10 min for recreational activities (separately for moderate- and vigorous-intensity activities), walking/bicycling for transportation, and work (separately for moderate- and vigorous-intensity activities). Work included both paid and unpaid work, household chores, and yard work; participants were instructed to think of work as “the things that you have to do such as paid or unpaid work, household chores, and yard work.” Therefore occupational and domestic physical activity could not be assessed separately. For the active transportation domain, information on all walking and bicycling are collected; however, these activities are not differentiated nor is the intensity level assessed [13]. As in other studies [15], walking/bicycling for transportation was assumed in our study to be of moderate intensity. This is in accordance with the WHO GPAQ analysis guide which assigns a metabolic equivalent of task (MET) of 4 to the active transportation domain [16]. The most extensive study of the validity and reliability of the GPAQ was conducted among 2657 adults from nine diverse countries, particularly among countries with lower educational levels [17]. Criterion validity was assessed using objective motion monitors, either a pedometer or accelerometer. Test-retest reliability was examined using a 3- to 7-day time gap between data collection. The results showed that the GPAQ performed well. The level of pooled criterion validity (from pedometer step counts) for the GPAQ assessed total physical activity score was fair (*r* = 0.31), but was lower for total vigorous-intensity physical activity compared with average vigorous activity counts/day from an accelerometer (*r* = 0.23 to *r* = 0.26). Test-retest reliability data produced good-to-excellent results, indicating a high level of repeatability between administrations (*r* = 0.67–0.81) [17].

We calculated the minutes/week spent in moderate- and vigorous-intensity activities in each of the three domains (recreational; active transportation; work). We also calculated an overall (total) measure of aerobic activity, comprising minutes/week spent in moderate-to-vigorous intensity physical activity (MVPA) across all domains. Participants were classed as aerobically active if they reached the recommended 150 min/week of moderate-intensity physical activity, 75 min/week of vigorous-intensity physical activity, or a combination of the two [18]. The equivalent binary indicator was calculated for each domain.

Highest educational attainment was obtained among adults aged 20 years and older, and was categorized as follows: less than high school (≤11th grade, but includes 12th grade with no diploma); high school diploma/general education development (GED) test; some college or associate (AA) degree; and college graduate or higher. Categories were created for age (20–39, 40–59, ≥60 years), and race/ethnicity (Non-Hispanic white, Hispanic/Mexican, Non-Hispanic black, other). Participants reported race/ethnicity from a list provided to them that included an open-ended response.

### Statistical analyses

Our analyses were limited to *N* = 29,201 adults aged 20 years and older, consistent with the reference age of the questions used to measure educational attainment. 39 adults (0.1%) were excluded from our complete-case analyses due to missing education data; 123 adults with valid education data were excluded due to missing physical activity data (0.4%). This resulted in an analytical sample of *N* = 29,039 adults with valid data for educational attainment and each physical activity indicator.

Highest educational attainment was cross-tabulated with age, gender, and race/ethnicity. Analyses were conducted separately for each domain and for overall (total) MVPA. Bivariate analysis consisted of the chi-square χ2 test for differences in the proportion of adults categorized as active (≥150 min/week) by age, gender, and race/ethnicity. Regression analyses were conducted on the data pooled over the five, 2-year NHANES survey cycles to assess potential independent and interactive relations of educational group, gender, age, and race/ethnicity to each physical activity indicator. Logistic regression was used since the distribution of the outcomes (inclusion of zeros and right-skew) precluded modelling the minutes/week spent active in continuous form [19, 20]. To explore possible moderation of the associations between education and physical activity we included each two-way interaction term (education × gender; education × age; education × race/ethnicity), in addition to the first-order terms of education, gender, age, race/ethnicity, and survey cycle. These variables were entered in the models as categorical terms with the following reference categories: education (less than high school); age (20–39 years); gender (male); and race/ethnicity (Non-Hispanic white). Wald tests were used to formally test the two-way interaction terms. Estimates of association are presented on the absolute scale since differences in activity prevalence between groups may distort comparisons of effect sizes expressed on the relative scale (e.g., through odds ratios)—an identical absolute difference in activity prevalence would have a larger odds ratio if activity prevalence was lower [21]. Our approach is consistent with calls for increasing use of absolute difference measures in public health research [22].

Prevalence estimates and 95% confidence intervals (95% CI) were calculated using model-based predictive margins [23]. Firstly, the proportion of adults categorized as active was estimated for each educational group after adjustment for age, gender, and race/ethnicity. Secondly, estimates for each moderator by educational group were mutually-adjusted (e.g., % active for each age category by education was adjusted for gender and race/ethnicity). Estimates for subgroups included adults classified in the ‘other’ race/ethnic group but are not reported separately given their small sample size and likely heterogeneity. To facilitate interpretation of the possible moderation of education-related disparities in physical activity, we show graphically the absolute difference in the estimated probability of being active for each educational group versus the reference (less than high school) for each level of the moderator, after mutual adjustment for the other variables.

To examine if physical activity disparities changed across time (2007 to 2016), we tested two-way interaction terms between education and year (linear term). Three-way interaction terms were also tested to examine if changes across year differed in demographic sub-groups (education × gender × year; education × age × year; education × race/ethnicity × year). The NHANES survey cycle (year) was entered into the models as a continuous variable (range 1–5).

All estimates were weighted using the 2-year sample weights provided by NHANES to account for the differential sample selection, survey nonresponse and post-stratification adjustments. Analyses were performed in Stata, version 15.0 (StataCorp LP, College Station, Texas).

### Sensitivity analyses

To investigate if changes in the distribution of education across time affected our findings, we repeated our analyses by converting the categories of highest educational attainment into a ridit score. Estimates from the resulting model can be interpreted as the Slope Index of Inequality [24]. To examine if the analyses were sensitive to the specific cut-point used for overall MVPA, alternative cut-points were used (≥60, ≥90 and ≥ 120 min/week in overall MVPA).

## Results

Age, gender and race/ethnicity were strongly associated with highest educational attainment: the proportion of adults achieving some college / associate degree or higher was lowest amongst older adults, and was highest amongst women and Non-Hispanic whites (*P* for χ^2^-tests all < 0.001; see Additional file 1).

### Leisure-time

The proportion of adults categorized as active (≥150 min/week) in the leisure-time domain differed by age, gender and race/ethnicity: activity levels were highest amongst younger adults, men, and Non-Hispanic whites (*P* for χ^2^-tests all < 0.001; see Additional file 2).

**Fig. 1:**
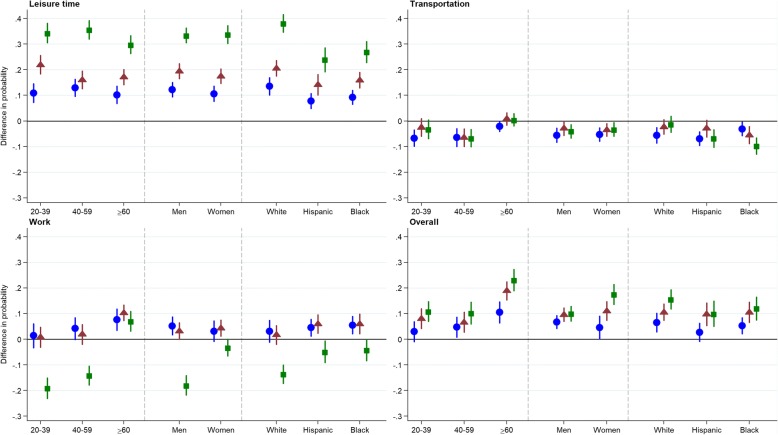
Estimated differences in physical activity outcomes by educational group, stratified by age, gender, and race/ethnicity. Estimated differences in physical activity outcomes (95% CI) according to highest educational attainment, stratified by age, gender, and race/ethnicity among US adults aged 20+ years, National Health and Nutrition Examination Survey, 2007–16. Education groups are: less than high school (referent), high school graduate (circles), some college (triangles), and college graduate (squares)

Activity was highest amongst highly-educated adults, similarly from 2007 to 2016 (education × year: *P* = 0.908) (Table 1). However, the magnitude of these education-related disparities was largest amongst younger adults (education × age: *P* = 0.016) and Non-Hispanic whites (education × ethnicity: *P* = 0.002) (Table 1 and Fig. 1). Differences in the magnitude of education-related disparities according to race/ethnicity were particularly pronounced. For example, the estimated difference in the probability of spending ≥150 min/week in leisure-time activity between adults in the highest versus the lowest educational groups was 38.1% (95% CI: 34.8, 41.4) amongst Non-Hispanic whites and 23.9% (95% CI: 19.3, 28.4) amongst Hispanic/Mexicans; a 14.2%-point or over 1.5-fold difference (Fig. 1). Moderation of these education-related disparities persisted over the 10-year study period (education × year × demographic sub-group: *P* all > 0.05).

**Table 1:** Probabilities of spending ≥150 min/week in moderate-to-vigorous physical activities amongst 29,039 US adults aged ≥20 years

Physical activity domain, education group	All	Age group			Gender		Race/ethnicity		
		20–39	40–59	≥60	Men	Women	White	Hispanic	Black
	% (SE)	%^a^ (SE)	%^a^ (SE)	%^a^ (SE)	%^a^ (SE)	%^a^ (SE)	%^a^ (SE)	%^a^ (SE)	%^a^ (SE)
Leisure-time									
< 11th grade	18.4 (0.8)	26.5 (1.4)	15.4 (1.1)	10.9 (0.8)	22.1 (1.0)	14.9 (1.0)	17.2 (1.2)	20.8 (1.0)	19.1 (1.1)
High school	29.7 (0.9)	37.4 (1.3)	28.2 (1.4)	21.1 (1.4)	34.3 (1.1)	25.5 (1.1)	30.6 (1.2)	28.6 (1.4)	28.3 (1.3)
Some college	36.7 (0.9)	48.4 (1.3)	31.4 (1.4)	28.0 (1.3)	41.5 (1.1)	32.3 (1.1)	37.7 (1.1)	34.9 (1.5)	35.0 (1.1)
College graduate or higher	51.9 (1.0)	60.8 (1.3)	50.9 (1.4)	40.6 (1.6)	55.4 (1.1)	48.6 (1.4)	55.3 (1.2)	44.6 (2.1)	46.0 (1.6)
*P* education x year	0.908								
*P* education x demographic group		0.016			0.253		0.002		
*P* education x demographic group x year		0.341			0.384		0.535		
Active transportation									
< 11th grade	16.4 (0.9)	19.7 (1.3)	17.6 (1.4)	9.6 (0.7)	18.4 (1.1)	14.5 (1.0)	14.7 (1.2)	18.3 (1.4)	19.3 (1.3)
High school	10.9 (0.7)	13.0 (1.0)	11.1 (0.9)	7.5 (0.8)	12.8 (0.8)	9.2 (0.8)	9.1 (0.8)	11.5 (1.2)	16.2 (1.1)
Some college	13.2 (0.7)	17.2 (1.3)	11.0 (0.8)	10.4 (0.9)	15.5 (0.8)	11.0 (0.8)	12.4 (0.9)	15.4 (1.0)	13.8 (1.1)
College graduate or higher	12.7 (0.8)	16.5 (1.4)	10.9 (0.8)	10.0 (1.1)	14.3 (0.8)	11.2 (0.9)	13.4 (1.0)	11.5 (1.3)	9.6 (0.9)
*P* education x year	0.111								
*P* education x demographic group		0.006			0.737		< 0.001		
*P* education x demographic group x year		0.103			0.168		0.334		
Work									
< 11th grade	36.9 (1.0)	47.3 (1.7)	39.3 (1.5)	19.0 (1.1)	46.0 (1.4)	28.3 (1.3)	41.5 (1.4)	31.1 (1.1)	29.0 (1.3)
High school	41.0 (1.0)	48.7 (1.5)	43.5 (1.5)	26.6 (1.7)	51.3 (1.1)	31.4 (1.3)	44.6 (1.3)	35.5 (1.5)	34.5 (1.4)
Some college	40.6 (0.8)	48.1 (1.1)	41.2 (1.4)	29.3 (1.3)	49.2 (1.1)	32.6 (0.9)	43.2 (1.0)	37.0 (1.5)	35.0 (1.3)
College graduate or higher	26.5 (0.8)	28.2 (1.4)	25.2 (1.1)	26.0 (1.6)	28.1 (1.5)	25.1 (0.9)	27.9 (1.1)	26.2 (1.9)	24.8 (1.5)
*P* education x year	0.519								
*P* education x demographic group		< 0.001			< 0.001		0.021		
*P* education x demographic group x year		0.951			0.633		0.497		
Overall (total) MVPA									
< 11th grade	54.7 (1.0)	67.4 (1.4)	56.8 (1.5)	33.8 (1.3)	63.1 (1.1)	47.0 (1.4)	56.0 (1.4)	53.0 (1.4)	50.3 (1.5)
High school	60.3 (0.9)	70.4 (1.2)	61.5 (1.5)	44.3 (1.6)	69.9 (0.9)	51.5 (1.4)	62.6 (1.1)	55.7 (1.8)	55.6 (1.2)
Some college	65.1 (0.8)	75.4 (1.1)	63.4 (1.3)	52.8 (1.4)	72.8 (1.0)	58.0 (1.1)	66.6 (1.0)	62.8 (1.5)	60.8 (1.3)
College graduate or higher	68.6 (0.9)	78.2 (1.2)	67.0 (1.2)	57.0 (1.6)	73.1 (1.1)	64.5 (1.2)	71.7 (1.1)	63.0 (2.2)	62.3 (1.6)
*P* education x year	0.440								
*P* education x demographic group		< 0.001			< 0.001		0.222		
*P* education x demographic group x year		0.185			0.793		0.494		

Abbreviations: *SE* standard error

^a^Prevalence estimates are mutually adjusted (e.g., estimates for age group by education strata adjusted for gender and ethnicity). Hispanic also includes Mexican

### Active transportation

Levels of active transportation (≥150 min/week) differed by age, gender and race/ethnicity: activity levels were highest in younger adults and in men, and were lowest amongst Non-Hispanic whites (*P* for χ2-tests all < 0.001; Additional file 2).

Amongst the educational groups, active transportation was highest in lower educated adults, similarly from 2007 to 2016 (education × year: *P* = 0.111). Education-related disparities were weak in magnitude. For example, the estimated probability of spending ≥150 min/week in active transportation was 16.4% (95% CI: 14.5, 18.2) amongst adults with less than high-school education compared with 12.7% (95% CI: 11.2, 14.2) of college graduates or higher (Table 1). Education-related disparities in active transportation were larger amongst younger- and middle-aged adults (education × age: *P* = 0.006), and amongst Hispanic/Mexicans and Non-Hispanic blacks (education × ethnicity: *P* < 0.001), but showed no difference by gender (education × gender: *P* = 0.737). However, these associations did not systematically differ over the 10-year study period (education × year × demographic sub-group: *P* all > 0.10).

### Work

The proportion of adults classed as active in the work domain (≥150 min/week) differed by age, gender and race/ethnicity: activity levels were highest amongst younger adults, men, and Non-Hispanic whites (*P* for χ2-tests all < 0.001; Additional file 2).

Highest educational attainment was non-linearly related to work-based physical activity—it was highest amongst adults with high school or some college education, and was lowest amongst college graduates or higher. These disparities did not systematically differ from 2007 to 2016 (education × year: *P* = 0.519).

Education-related disparities in work-based activity were larger amongst younger compared with older adults (education × age: *P* < 0.001); men compared with women (education × gender: *P* < 0.001); and amongst Non-Hispanic whites (education × ethnicity: *P* = 0.021; Table 1 and Fig. 1). For example, the estimated difference in the probability of spending ≥150 min/week in work-based activity between adults in the lowest versus the highest educational groups was 18.0% (95% CI: 14.3, 21.7) amongst men and 3.3% (95% CI: 0.0, 6.5) amongst women; a 14.7%-point or over 5-fold difference (Fig. 1). However, these associations did not systematically differ over the 10-year study period (education × year × demographic sub-group: *P* all > 0.10).

### Overall (total) MVPA

The proportion of adults classed as aerobically active (MVPA ≥150 min/week) across all domains differed by age, gender and race/ethnicity: activity levels were highest amongst younger adults, men, and Non-Hispanic whites (*P* for χ^2^-tests all < 0.001; Additional file 2).

Activity levels were highest amongst highly-educated adults, similarly from 2007 to 2016 (education × year: *P* = 0.440). The estimated probability of spending ≥150 min/week in MVPA was 68.6% (95% CI: 66.9, 70.3) amongst college graduates or higher compared with 54.7% (95% CI: 52.7, 56.8) of adults with less than high-school education (Table 1). However, the magnitude of these education-related disparities were larger amongst older compared with younger adults (education × age: *P* < 0.001), and amongst women compared with men (education × gender: *P* < 0.001; Table 1 and Fig. 1). For example, the estimated difference in the probability of being aerobically active between adults in the highest versus the lowest educational groups was 23.1% (95% CI: 19.1, 27.2) amongst those aged ≥60 years, and 10.8% (95% CI: 7.1, 14.6) amongst those aged 20–39 (Fig. 1); a 12.3%-point or over 2.1-fold difference. The corresponding difference was 17.5% (95% CI: 13.7, 21.3) amongst women, and 10.0% (95% CI: 7.2, 12.7) amongst men (Fig. 1).

### Sensitivity analyses

Accounting for the change in the distribution of educational attainment over the 10-year period by using the SII yielded similar findings (see Additional file 3 and Additional file 4). Findings were also similar when using alternative cut-off points for classifying participants as sufficiently active (≥60, ≥90 and ≥ 120 min/week in overall MVPA; see Additional file 5).

## Discussion

Using nationally-representative data from the US, we found that levels of active participation in moderate-to-vigorous intensity physical activity across all domains were highest amongst highly-educated adults. However, the direction and the magnitude of education-related disparities differed by domain. Amongst highly-educated adults, active participation was higher for leisure-time activities, but lower for the active transportation and work domains. The magnitude of these disparities was in some cases greatly moderated by age, gender, and race/ethnicity—magnitudes of disparity between the highest- and lowest-educated groups differed by up to 5-fold between these sub-groups. The net result of this moderation was that overall (total) activity disparities according to education group were larger in older adults and amongst women.

Our findings are in agreement with the few studies which have investigated how education-related disparities in leisure-time physical activity change across time [6, 7]—these showed persisting inequalities (in descriptive terms) from 2000 to 2009 and (in relative terms) from 1990 to 2004 [7]. We add to previous findings by providing a detailed and contemporaneous assessment of physical activity disparities, and documenting their persistence up to 2016. Our analysis showed contrasting disparities for recreational and non-recreational physical activity (active transportation and work), yet the net result showed that highly-educated adults had higher overall levels of aerobic physical activity than their lower-educated counterparts.

We also documented the moderation of education-related disparities in physical activity by age and race/ethnicity in each domain, and by gender for work-based activity. Our findings suggest that the intermediary drivers of such disparities—differences in financial resources [3, 4], health status [25], psychological or cultural characteristics [25, 26], and environmental conditions [25, 27, 28]—may differ across these demographic groups and that these differences exist within the same level of educational attainment [29] which in turn moderates the magnitude of the disparities in physical activity.

Education-related disparities in leisure-time activity varied by race/ethnicity—this was driven by the substantially higher prevalence of recreational activity amongst Non-Hispanic white college graduates. Three plausible mechanisms may explain this finding. First, racial/ethnic disparities in other socioeconomic factors which exist within the same level of educational attainment may affect levels of leisure-time activity. For example, income and wealth returns to college education have been shown to be larger amongst whites [30, 31], potentially leading to better access and affordability for their leisure-time participation than those experienced by college graduates in other racial/ethnic groups [32]. Second, racial/ethnic disparities in health also exist within the same level of education attainment [33]—as such, the higher leisure-time activity participation amongst Non-Hispanic white college graduates may also be partially attributable to a lower prevalence of activity-limiting health conditions (including mobility disability). Third, built environmental factors may also play a role—factors which influence the opportunities to undertake recreational activity (e.g., neighborhood safety, public green space availability) may impact on activity participation, and due to segregation differ by race/ethnicity even amongst persons within the same education group [34–38].

Education-related disparities in leisure-time activity were also marginally larger amongst younger compared with older adults. Younger highly-educated adults may be more active in sports and other recreational activities than their highly-educated older counterparts, potentially due to differences in lifestyle aspirations, physical health, or the effectiveness of public health campaigns [39].

Disparities in active transportation, favoring the lowest-educated groups, possibly reflect fewer financial resources for car ownership [40]. Education-related disparities in active transportation also differed by age. Among persons aged 20–39, active transportation was highest amongst the lowest-educated; among persons aged ≥60, active transportation was marginally higher amongst college graduates. Disparities also differed by race/ethnicity—while active transportation was lowest amongst all college graduates, it was especially low amongst Non-Hispanic blacks. Further work is required to understand the extent to which the education-related disparities in active transportation (e.g., walking and bicycling) found in our study are driven by policy modifiable factors—while commute distances are challenging to modify, modifiable barriers to active transportation may include psychological factors such as neighborhood safety [41], or knowledge/response to public health messages highlighting the health benefits of active travel.

Disparities in work-based physical activity, which for the purposes of the present study included ‘paid or unpaid work’, ‘household chores’ and ‘yard work’, differed by age, gender, and race/ethnicity—levels of work-based physical activity were higher amongst the lowest-educated groups, and these differences were larger amongst younger adults, men, and Non-Hispanic whites. These differences are likely to be partly attributable to differences in the distribution of physically active occupations. Engagement in strenuous job-related activities and household chores are more likely to be undertaken by adults with low levels of educational attainment [42], who are also male and of younger age. Amongst the lower-educated groups, the higher levels of work-based activity for Non-Hispanic whites may reflect racial/ethnic differences in employment levels during and post the Great Recession [43, 44] rather than differences in levels of work-based physical activity per se.

Strengths of our study include the use of NHANES data which enables nationally-representative inference, and a more detailed investigation of education-related disparities across multiple domains of physical activity than in previous studies, or in alternative datasets [8]. Although we demonstrated that education-related disparities differ across the leisure-time, active transportation, and work domains, we were unable to examine more detailed types of activities which may be of particular interest. For example, education-related disparities may be largest for formal leisure-time activities that require greater financial resources to undertake. The questionnaire used (GPAQ) does not capture information on the level of intensity for walking/bicycling for transportation, nor separate these different activities; this may be a useful distinction to make in future studies, given the potentially different determinants of these activities.

These data also enabled investigation of the moderation of education-related disparities in physical activity according to age, gender, and race/ethnicity. However, while NHANES is a comparatively large sample, it is potentially underpowered to detect interaction terms which typically require very large sample sizes [45, 46]. Our study is unlikely therefore to provide definitive evidence on the presence of modest yet health-impacting education-related disparities in physical activity, and their change over time, across age, gender, or racial/ethnic sub-groups. Other factors which may plausibly moderate physical activity disparities include area of residence, which is likely to impact on both physical activity and economic opportunity [34–38, 47].

The use of identical instruments over five, 2-year NHANES survey cycles enabled the investigation of trends across time, though the availability of comparable data limited the timespan of investigation from 2007 onwards [14]. Reassuringly, we found that results were robust to the use of different cut-offs for classifying overall (total) moderate-to-vigorous intensity physical activity, and the use of the Slope Index of Inequality to account for differences in the distribution of educational attainment across time. However, as in all studies examining trends in disparities, interpretation may be affected by changes in the selection of individuals into the different educational groups over the study period [48]. Due to differences in selection and potential causal effects, results may differ when using alternative indicators of socioeconomic status—this warrants future investigation.

Self-reported physical activity is subject to recall and desirability biases, which may lead to the misclassification of activity status [49, 50]. Such misclassification may differ by education and/or demographic sub-group, potentially upwardly or downwardly biasing our estimates of disparities in physical activity. Furthermore, differences in the perception of physical activity questions across demographic sub-groups (e.g., in what constitutes ‘moderate’ or ‘vigorous’ activity) [51] might also lead to artefactual differences in the magnitude of physical activity disparities. Investigation of trends over time in objectively measured physical activity may be valuable in future monitoring efforts, although this may be challenging since activity monitors do not currently capture data on activity domain, and current public health guidelines for aerobic activity are based on self-reported data.

Finally, causation cannot be straightforwardly inferred from these observational data. Specifically, while classifying participants according to educational attainment may be beneficial—alternative indicators such as income and occupation may be more likely affected by reverse causation [52] or confounding due to ill health—education-related disparities in physical activity may be attributable to other correlated socioeconomic factors, or preceding parental characteristics which may influence both educational attainment and physical activity levels.

Notwithstanding these caveats, our findings may have implications for both policy and related research. First, our finding documenting the persistence of education-related disparities in leisure-time physical activity over the past 10 years suggests that previous policies have not been sufficient, and that additional policy initiatives are required. Second, our findings suggest that in order to reduce disparities in physical activity, there may be sub-groups of the population that may benefit most from intervention—those in which the education-related disparities were largest. Such initiatives may thereby indirectly lead to a narrowing of physical activity disparities (and thereby health disparities) by age, gender, and race/ethnicity. Finally, our findings highlight the importance of considering the domain-specific nature of physical activity disparities. The contrasting disparities for recreational and non-recreational physical activity (active transportation and work), favoring the highest- and lowest-educated groups respectively, are especially worrisome as it is increasingly recognized that whilst taking part in recreational activities is beneficial for physical- and mental-health, high levels of involvement in occupational physical activity can be detrimental for health [53–55].

## Conclusions

In conclusion, the large education-related disparities in recreational activity, in favor of the highest-educated groups, outweighed the opposite pattern for non-recreational activities (active transportation and work). Our results suggest that addressing socioeconomic disparities in physical activity requires the assessment of multiple domains of activity, and identification of the different demographic sub-groups for which these disparities are more or less pronounced. These findings should be considered in the future monitoring of socioeconomic disparities in physical activity or of other health-related outcomes, and in the identification of sub-groups that would benefit most from targeted interventions.

## Additional files

Additional file 1: Highest educational attainment by demographic subgroups (age, gender, and race/ethnicity). (DOCX 41 kb)

Additional file 2: Activity levels (≥150 min/week) by demographic subgroups (age, gender, and race/ethnicity). (DOCX 42 kb)

Additional file 3: Differences in activity levels by demographic subgroups (age, gender, and race/ethnicity) using the Slope Index of Inequality. (DOCX 23 kb)

Additional file 4: Differences in activity levels by demographic subgroups (age, gender, and race/ethnicity) using the Slope Index of Inequality. (TIF 118 kb)

Additional file 5: Estimated differences in % active using different cut-offs (≥60, ≥90 and ≥ 120 min/week in overall MVPA) by educational group, stratified by age, gender, and race/ethnicity. (TIF 136 kb)
