# Supplementary material for: Education-related disparities in reported physical activity during leisure-time, active transportation, and work among US adults: repeated cross-sectional analysis from the National Health and Nutrition Examination Surveys, 2007 to 2016
Source: BMC Public Health. 2018 Jul 28;18:926. doi: 10.1186/s12889-018-5857-z (PMC6064072; doi:10.1186/s12889-018-5857-z)
Supplement: Supplementary file 1 — Highest educational attainment by demographic subgroups (age, gender, and race/ethnicity). (DOCX 41 kb) [file 12889_2018_5857_MOESM1_ESM.docx]

**Additional file 1.** Highest educational attainment by demographic sub-groups (age, gender, and race/ethnicity) amongst 29,039 US adults aged ≥20years, National Health and Nutrition Examination Survey, 2007-16^a^

| **Demographics** | **Highest Educational Attainment** | | | | **P-value^b^** |
| --- | --- | --- | --- | --- | --- |
|  | **<11^th^ grade** | **High school** | **Some college** | **College graduate or higher** |  |
|  | **N (%)** | **N (%)** | **N (%)** | **N (%)** |  |
| **All** | 7541 (17.1) | 6563 (22.2) | 8327 (31.3) | 6608 (29.4) |  |
| **Age group:** |  |  |  |  |  |
| 20-39 | 1967 (15.6) | 2206 (21.2) | 3290 (34.9) | 2350 (28.3) | <0.001 |
| 40-59 | 2432 (16.6) | 2123 (22.0) | 2632 (29.5) | 2348 (31.9) |  |
| ≥60 | 3142 (20.0) | 2234 (23.8) | 2405 (28.9) | 1910 (27.3) |  |
|  |  |  |  |  |  |
| **Gender:** |  |  |  |  |  |
| Men | 3784 (17.9) | 3343 (23.4) | 3691 (29.0) | 3256 (29.7) | <0.001 |
| Women | 3757 (16.3) | 3220 (21.0) | 4636 (33.5) | 3352 (29.1) |  |
|  |  |  |  |  |  |
| **Race/ethnicity:** |  |  |  |  |  |
| Non-Hispanic white | 1887 (11.2) | 2936 (22.5) | 3818 (32.6) | 3306 (33.6) | <0.001 |
| Hispanic/Mexican | 3544 (42.4) | 1517 (21.3) | 1690 (24.5) | 832 (11.8) |  |
| Non-Hispanic black | 1473 (21.5) | 1611 (26.0) | 2023 (34.7) | 1037 (17.8) |  |

^a^ Sample sizes are unweighted numbers; row %’s are weighted to reflect NHANES complex, multistage survey sampling

^b^ *P* value for comparison between education groups, calculated by the χ^2^ test
