# Supplementary material for: Education-related disparities in reported physical activity during leisure-time, active transportation, and work among US adults: repeated cross-sectional analysis from the National Health and Nutrition Examination Surveys, 2007 to 2016
Source: BMC Public Health. 2018 Jul 28;18:926. doi: 10.1186/s12889-018-5857-z (PMC6064072; doi:10.1186/s12889-018-5857-z)
Supplement: Supplementary file 2 — Activity levels (≥150 min/week) by demographic subgroups (age, gender, and race/ethnicity). (DOCX 42 kb) [file 12889_2018_5857_MOESM2_ESM.docx]

**Additional file 2.** Activity levels (% ≥150 minutes/week) by age, gender, and race/ethnicity amongst 29,039 US adults aged ≥20years, National Health and Nutrition Examination Survey, 2007-16^a^

| **Demographics** | **N** | **Physical activity domain** | | | |
| --- | --- | --- | --- | --- | --- |
|  |  | **Leisure-time** | **Active transportation** | **Work** | **Overall MVPA** |
|  |  | **% active (SE)** | **% active (SE)** | **% active (SE)** | **% active (SE)** |
| **All** | 29,039 | 37.1 (0.8) | 13.3 (0.5) | 35.5 (0.6) | 63.3 (0.6) |
| **Age group:** |  |  |  |  |  |
| 20-39 | 9813 | 46.5 (1.0) | 17.0 (0.9) | 41.8 (0.8) | 73.8 (0.7) |
| 40-59 | 9535 | 34.8 (1.0) | 12.4 (0.6) | 36.2 (0.8) | 63.4 (0.8) |
| ≥60 | 9691 | 27.0 (0.9) | 9.3 (0.5) | 25.5 (0.9) | 48.4 (1.0) |
| *P-value* |  | *<0.001* | *<0.001* | *<0.001* | *<0.001* |
|  |  |  |  |  |  |
| **Gender:** |  |  |  |  |  |
| Men | 14,074 | 41.3 (0.9) | 15.4 (0.6) | 42.9 (0.9) | 71.0 (0.6) |
| Women | 14,965 | 33.2 (0.9) | 11.3 (0.6) | 28.7 (0.5) | 56.3 (0.8) |
| *P-value* |  | *<0.001* | *<0.001* | *<0.001* | *<0.001* |
|  |  |  |  |  |  |
| **Race/ethnicity:** |  |  |  |  |  |
| Non-Hispanic white | 12,047 | 39.0 (1.1) | 11.9 (0.6) | 37.3 (0.8) | 65.1 (0.8) |
| Hispanic/Mexican | 7583 | 31.5 (0.7) | 16.5 (1.0) | 35.8 (0.9) | 61.0 (1.0) |
| Non-Hispanic black | 6144 | 32.5 (0.8) | 15.0 (0.9) | 32.0 (0.7) | 58.1 (0.8) |
| *P-value* |  | *<0.001* | *<0.001* | *<0.001* | *<0.001* |

SE: standard error

^a^ Sample sizes are unweighted numbers; row %’s are weighted to reflect NHANES complex, multistage survey sampling

^b^ *P* value for comparison in the % active between demographic groups, calculated by the χ^2^ test
