# Supplementary material for: Education-related disparities in reported physical activity during leisure-time, active transportation, and work among US adults: repeated cross-sectional analysis from the National Health and Nutrition Examination Surveys, 2007 to 2016
Source: BMC Public Health. 2018 Jul 28;18:926. doi: 10.1186/s12889-018-5857-z (PMC6064072; doi:10.1186/s12889-018-5857-z)
Supplement: Supplementary file 3 — Differences in activity levels by demographic subgroups (age, gender, and race/ethnicity) using the Slope Index of Inequality. (DOCX 23 kb) [file 12889_2018_5857_MOESM3_ESM.docx]

**Additional file 3.** Probabilities of spending ≥150minutes/week in moderate-to-vigorous physical activities amongst 29,039 US adults aged ≥20 years using the Slope Index of Inequality

| **Physical activity domain,**  **education group** |  | **Age group** |  |  | **Gender** |  | **Race/ethnicity** |  |  |
| --- | --- | --- | --- | --- | --- | --- | --- | --- | --- |
|  | All | 20-39 | 40-59 | ≥60 | Men | Women | White | Hispanic | Black |
| **Leisure-time** |  |  |  |  |  |  |  |  |  |
| Bottom | 16.0 | 24.7 | 12.3 | 8.9 | 20.4 | 11.9 | 14.8 | 18.7 | 17.5 |
| Top | 57.3 | 67.3 | 55.4 | 45.7 | 61.3 | 53.5 | 61.2 | 49.2 | 50.5 |
| SII | 41.3 | 42.6 | 43.1 | 36.7 | 40.9 | 41.6 | 46.4 | 30.6 | 33.0 |
| 95% CI | 38.1; 44.5 | 37.9; 47.3 | 38.3; 48.0 | 31.9; 41.6 | 37.3; 44.5 | 37.1; 46.2 | 42.1; 50.6 | 24.4; 36.8 | 28.3; 37.7 |
| *P* ridit score x year | *0.916* |  |  |  |  |  |  |  |  |
| *P* ridit score x demographic group | - | *0.111* |  |  | *0.770* |  | *<0.001* |  |  |
| *P* ridit score x demographic group x year | - | *0.465* |  |  | *0.510* |  | *0.188* |  |  |
| **Active transportation** |  |  |  |  |  |  |  |  |  |
| Bottom | 13.7 | 16.3 | 14.9 | 8.5 | 15.9 | 11.7 | 11.0 | 17.8 | 20.0 |
| Top | 12.3 | 16.6 | 9.3 | 10.5 | 14.0 | 10.7 | 13.3 | 10.8 | 7.9 |
| SII | -1.4 | 0.3 | -5.6 | 2.0 | -1.9 | -1.0 | 2.4 | -7.0 | -12.1 |
| 95% CI | -3.9; 1.1 | -4.1; 4.8 | -9.1; -2.1 | -1.2; 5.3 | -4.8; 1.0 | -3.8; 1.8 | -1.0; 5.8 | -11.7; -2.3 | -15.9; -8.2 |
| *P* ridit score x year | *0.062* |  |  |  |  |  |  |  |  |
| *P* ridit score x demographic group | - | *0.003* |  |  | *0.508* |  | *<0.001* |  |  |
| *P* ridit score x demographic group x year | - | *0.057* |  |  | *0.143* |  | *0.215* |  |  |
| **Work** |  |  |  |  |  |  |  |  |  |
| Bottom | 44.7 | 56.9 | 47.8 | 22.8 | 56.5 | 33.7 | 50.6 | 32.7 | 34.0 |
| Top | 27.9 | 29.1 | 25.8 | 29.2 | 29.6 | 26.3 | 27.7 | 32.6 | 28.9 |
| SII | -16.8 | -27.8 | -22.1 | 6.4 | -26.9 | -7.5 | -22.9 | 0.0 | -5.1 |
| 95% CI | -19.7; -13.9 | -32.4; -23.1 | -26.7; -17.4 | 1.6; 11.2 | -31.3; -22.5 | -11.2; -3.7 | -26.9; -18.8 | -5.4; 5.3 | -10.6; 0.5 |
| *P* ridit score x year | *0.534* |  |  |  |  |  |  |  |  |
| *P* ridit score x demographic group | - | *<0.001* |  |  | *<0.001* |  | *<0.001* |  |  |
| *P* ridit score x demographic group x year | - | *0.732* |  |  | *0.898* |  | *0.113* |  |  |
| **Total: recreation, active transportation, work** |  |  |  |  |  |  |  |  |  |
| Bottom | 54.8 | 66.9 | 56.9 | 34.4 |  |  |  |  |  |
| Top | 72.0 | 80.9 | 69.1 | 63.5 |  |  |  |  |  |
| SII | 17.2 | 14.0 | 12.2 | 29.0 |  |  |  |  |  |
| 95% CI | 14.0; 20.3 | 9.5; 18.4 | 7.0; 17.4 | 24.1; 33.9 |  |  |  |  |  |
| *P* ridit score x year | *0.665* |  |  |  |  |  |  |  |  |
| *P* ridit score x demographic group |  | *<0.001* |  |  | *<0.001* |  | *0.251* |  |  |
| *P* ridit score x demographic group x year |  | *0.144* |  |  | *0.359* |  | *0.250* |  |  |

Abbreviations: SE, standard error

**Notes:** The Slope Index of Inequality (SII) accounts for the entire distribution of the sample by educational group and also allows for the change in the relative size of the education groups over the 10-year period. To estimate the SII, we ranked the data by category of education (from lowest to highest) in each 2-year cycle and calculated for each group the midpoint of its position in the cumulative distribution of the population (ridit score). Linear regression models were used to regress the physical activity indicator on the ridit score (with age, gender, and race/ethnicity included in the model). Model-based marginal effects were calculated as in our main analysis to estimate the SII: the SII can be interpreted as the estimated absolute difference in the probability of being active (≥150 minutes/week) from the top of the cumulative education distribution to the bottom. Tests of the two-way interaction terms and tests of linear trend were calculated in the same way as described for the categories of educational attainment.
