# Supplementary figures and images for: Education-related disparities in reported physical activity during leisure-time, active transportation, and work among US adults: repeated cross-sectional analysis from the National Health and Nutrition Examination Surveys, 2007 to 2016
Source: BMC Public Health. 2018 Jul 28;18:926. doi: 10.1186/s12889-018-5857-z (PMC6064072; doi:10.1186/s12889-018-5857-z)

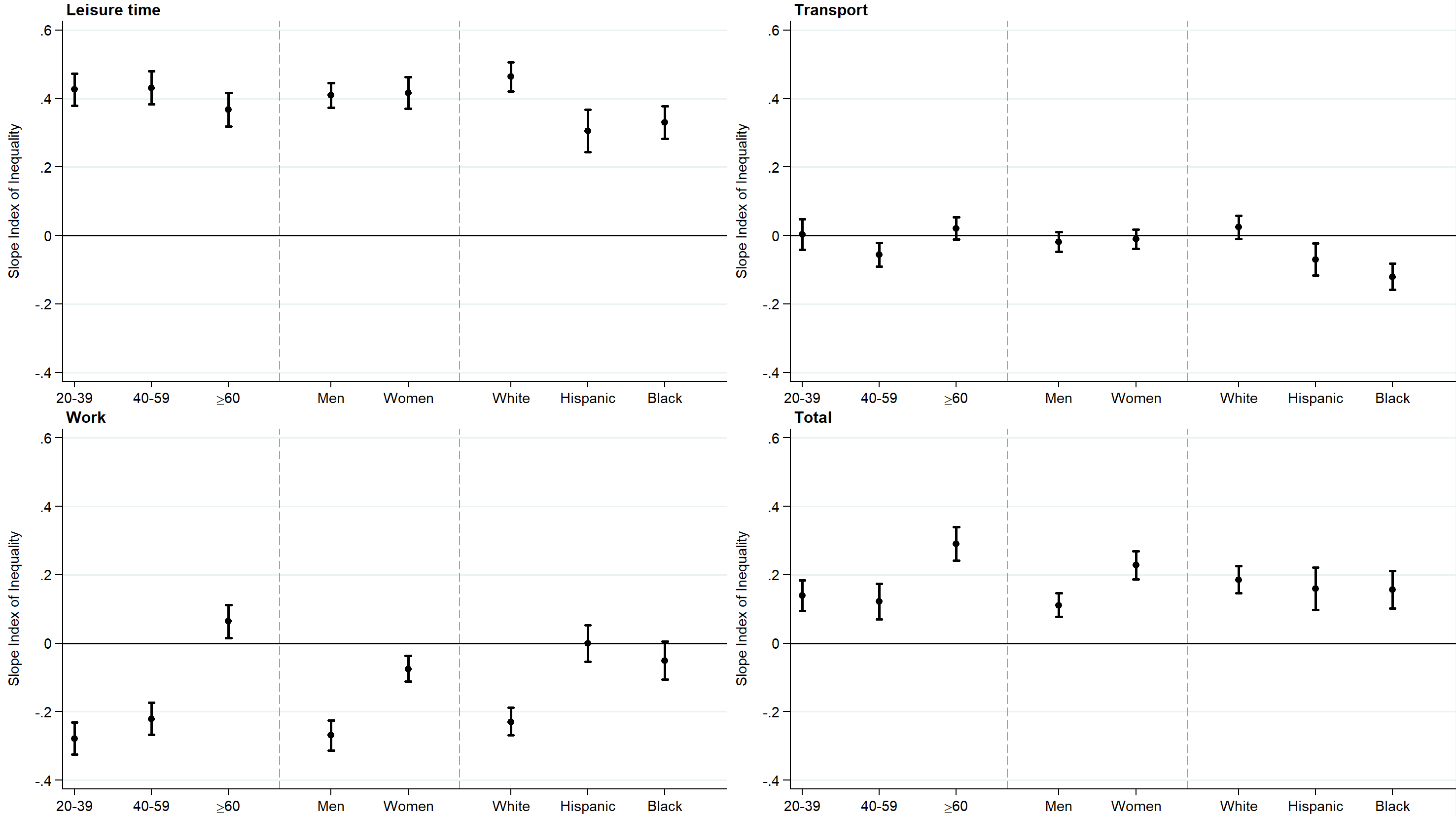

Supplement: Supplementary file 4 — Differences in activity levels by demographic subgroups (age, gender, and race/ethnicity) using the Slope Index of Inequality. (TIF 118 kb) [file 12889_2018_5857_MOESM4_ESM.tif]

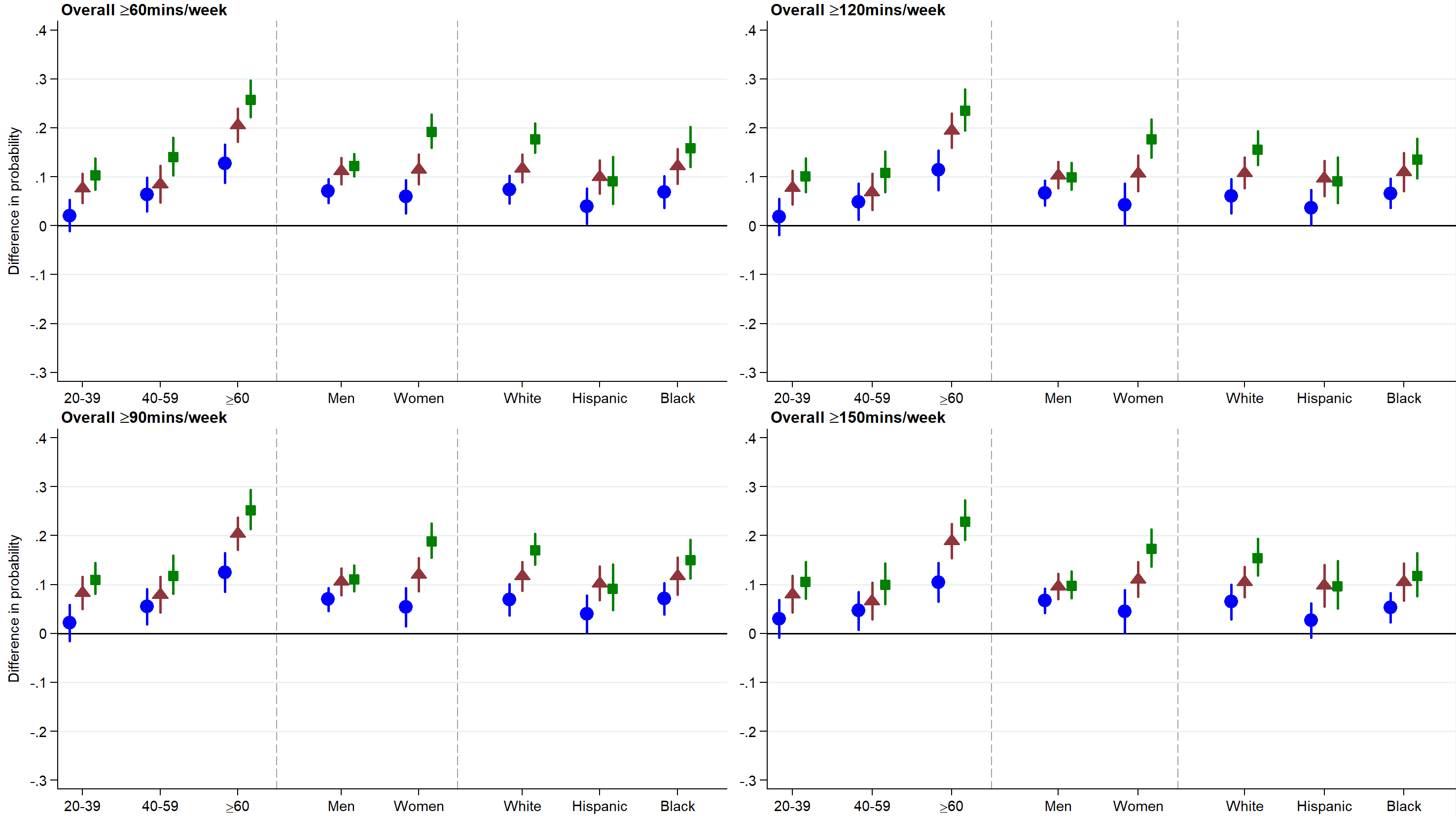

Supplement: Supplementary file 5 — Estimated differences in % active using different cut-offs (≥60, ≥90 and ≥ 120 min/week in overall MVPA) by educational group, stratified by age, gender, and race/ethnicity. (TIF 136 kb) [file 12889_2018_5857_MOESM5_ESM.tif]
